# Towards a better understanding of inequity and the psychological processes underlying the intergenerational transmission of socioeconomic status

**DOI:** 10.1016/j.socscimed.2024.117330

**Published:** 2024-09-12

**Authors:** Roy Otten, Thao Ha

**Affiliations:** a Radboud University Nijmegen, the Netherlands; b Arizona State University, USA

## Abstract

As poverty in the U.S. is increasing and the income gap continues to rise, addressing disparities in socioeconomic status (SES) has become a national priority. This study employs the Interactionist Model, a well-established theoretical framework for examining the intergenerational transmission of SES. Specifically, using longitudinal data from a sample of 998 adolescents, 47.2% of whom are females, from diverse ethnic backgrounds, we investigated how parents’ SES influences both their material and immaterial resources, and subsequently affects their offspring’s SES through inhibitory control during adolescence. Our findings support an indirect effect wherein parental SES influences the SES of the next generation via parental material and immaterial investments. Additionally, we demonstrate that immaterial investments influence the next generation’s SES, mediated by inhibitory control. The implications of these findings are further discussed.

## 1. Introduction

In 2023, the U.S. Census Bureau reported a troubling rise in child poverty, with approximately 9 million children, or 12.4% of all children, living in poverty in 2022. This marked a substantial increase from 5.2% in 2021 (Shrider and Creamer, 2022). Additionally, recent studies, including a 2020 report by the Pew Research Center, indicate that the income gap in the U.S. continues to widen, surpassing that of similar developed countries (Pew Research Center, 2020). Responding to these challenges, the National Academies of Sciences, Engineering, and Medicine highlighted in their 2023 report the urgent need to address SES inequities (National Academies of Sciences, Engineering, and Medicine, 2023). One important aspect underlying inequities in SES pertains to the transmission of SES from one generation to the next.

Among other aspects that are decisive for how transmission of SES takes place, the resources, opportunities, and education that is provided by parents are important. It is well-established that poverty and low SES limit children’s access to resources, opportunities, and education during their formative years in childhood and adolescence (Bradley and Corwyn, 2002). In contrast, children born into higher SES families with more wealth, education, and social standing enjoy life-long advantages in domains such as health (e.g., Savelieva et al., 2017), cognitive abilities (e.g., Campos-Vazquez, 2018), and socio-emotional well-being (e.g., Melchior et al., 2018; Bradley and Corwyn, 2002; Campos-Vazquez, 2018; Evans and Kim, 2013; Hackman and Farah, 2009). Although there is extensive research on the intergenerational transmission of SES, little is known about the underlying psychological processes (Evans and Kim, 2013). Understanding these processes is essential to breaking the intergenerational cycle of SES to inform interventions and policies that benefit future generations.

The Interactionist Model provides a well-established theoretical framework for understanding the intergenerational transmission of SES. This model highlights reciprocal influences and transactional processes between SES, family interactions, and individual development (Conger and Donnellan, 2007; Conger et al., 2010), accumulating personal, economic, and social disadvantages or advantages across time and generations. Central in the model is children’s access to parental material and immaterial resources. Children growing up in lower SES families have less access to parental material resources, such as income, safe neighborhoods, a stimulating home environment, and academic enhancing resources such as internet and computer facilities (Conger et al., 2010; Gershoff et al., 2007; Kaushal et al., 2011; Kalil and Ryan, 2020). Regarding parental immaterial resources, low SES negatively impacts parental functioning (Conger et al., 2021). Financial strain for instance can potentially lead to feelings of frustration, anger, and depression among parents. This emotional toll can diminish their ability to provide social support and deplete their emotional and physical vigor. Consequently, this situation is linked to less effective parenting behaviors, such as parental withdrawal, frustration, or hostility, as discussed by Dix and colleagues (2004). But the cycle of economic hardship, stress, and strained parenting also challenges parents’ ability to maintain a safe and secure environment (Brody et al., 2002; Gershoff et al., 2007; Linver et al., 2002; Mistry et al., 2008; Nievar and Luster, 2006). Thus, children in low-SES families have less access to supportive parenting and are more likely to encounter stressful family interactions (Bradley and Corwyn, 2002; Conger et al., 2021). These parental material and immaterial resources affect child behaviors and are ultimately responsible for the intergenerational transmission of SES.

Despite the link between parental SES and their material and immaterial investments, we know little about *how* these investments are related to SES of the next generation. This gap in our understanding prompts further investigation into the mechanisms that transmit socioeconomic advantages or disadvantages across generations. Recently, using the Interactionist Model, Conger and colleagues found that child and adolescent conscientiousness was fostered by a transactional process between higher family SES and parental material and immaterial investments (Conger et al., 2021). It is likely that there are other psychological mechanisms active in the transactional modell proposed by Conger and colleagues. In the present study we aim at testing the role of inhibitory control in the Interactionist Model. Inhibitory control, is the cognitive ability to control one’s impulses, emotions, or behaviors in the face of temptation, distraction, or conflicting information (Blakemore and Robbins, 2012; Barkley, 1997). It is a dynamic cognitive process that can be developed and improved through practice and training. Throughout adolescence, a noticeable shift towards more focused patterns of inhibitory control has been demonstrated, aligning gradually with the behavioral and neurological characteristics observed in adults (Casey et al., 2000). Inhibitory control is, therefore, more open to change and potentially a crucial intervention/prevention target for breaking the intergenerational transmission of SES. Previous research has shown that adolescents’ inhibitory control is one of the most robust predictors of lifelong adjustment and SES (Taylor and Barch, 2022). It leads to higher educational attainment and less antisocial behavior and substance use (e.g., Otten et al., 2019). Adolescence is a sensitive period for inhibitory control due to the interplay of brain maturation, hormonal changes, emotional sensitivity, and the societal context (e.g., Arain et al., 2013; Sowell et al., 1999; Dwyer et al., 2014) and it plays a critical role in these evolving social executive functions in adolescence and young adulthood (Crone et al., 2008; Ellis et al., 2004; Pharo et al., 2011; Vetter et al., 2013). However, inhibitory control has not been studied as an underlying mechanism of the intergenerational transmission of SES.

In sum, in this study we will expand on the work of Conger and colleagues by focusing on the role of inhibitory control within the Interactionist Model. Using longitudinal data that spans two generations, we expect that parents’ SES affects both parental material and immaterial investments, affecting their offspring’s SES via inhibitory control during late adolescence/young adulthood. By exploring the relationship between family SES, parental investments, inhibitory control, and SES of the next generation, we aim to gain a better understanding of the transactional processes between generations.

## 2. Method

### 2.1. Participants

The initial sample consisted of 998 adolescents and their families from a large randomized control trial of the Family Check-Up (FCU) intervention (Dishion and Stormshack, 2007). Participants were recruited from the sixth grade (age 11) of three public middle schools in the Pacific Northwest of the United States. In total, 90% of parents consented for their child’s participation. The Institutional Review Board at the University of Oregon approved the study procedures. The longitudinal retention was high; on average, 80% of those participants were assessed at baseline through age 27.

### 2.2. Procedure

**Fig. 1.**
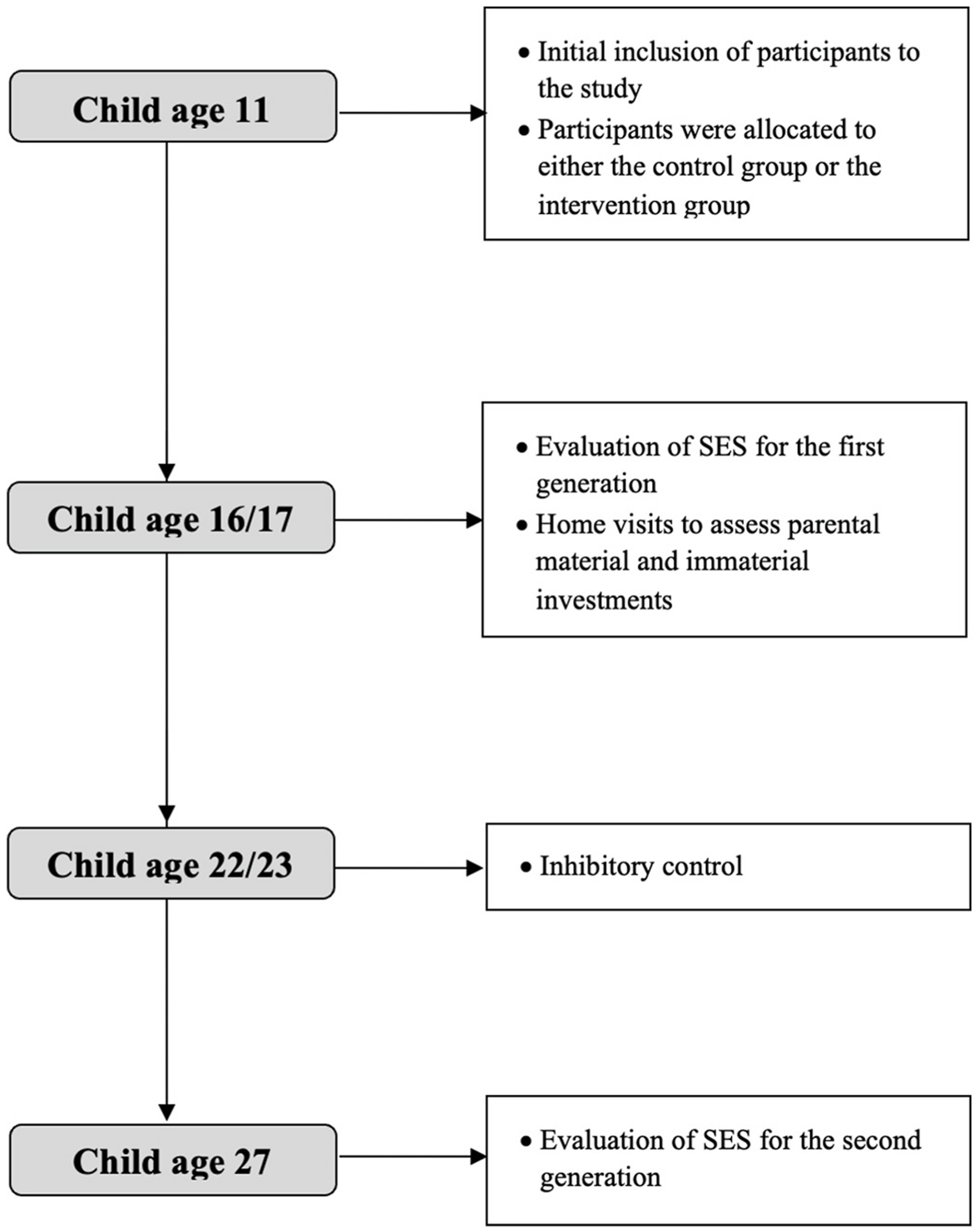
Flow diagram illustrating showing the different time points and assessed characteristics.

Participants were randomly assigned at the individual level to the control group (middle school as usual) or to a family-centered intervention. The intervention offered at-risk families a Family Check-Up (FCU). The Family Check-Up is a brief, strengths-based intervention for families with children. The intervention aims to improve parenting skills and family management practices, with the goals of improving a range of emotional, behavioral and academic child outcomes. The Family Check-Up consists of three main components: (1) an initial interview that involves rapport building and motivational interviewing to explore parental strengths and challenges related to parenting and the family context; (2) an ecological family assessment that includes parent and child questionnaires, a teacher questionnaire for children that are in school, and a videotaped observation of family interactions; and (3) tailored feedback that involves reviewing assessment results and discussing follow-up service options for the family. Follow-up services may include clinical or support services in the community. They may also include the Everyday Parenting program, which is a parenting management program that is typically delivered by the provider. Dishion and Kavanagh describe the FCU intervention more in detail (Dishion and Kavanagh, 2003). The FCU intervention effects are not the focus of this study and therefore the intervention group assignment was included as a covariate in the model. All respondents were assured of the confidentiality of their responses. Participants were compensated for their participation. Fig. 1 depicts flow diagram clarifying the different time points and assessments.

### 2.3. Measures

#### Socioeconomic Status.

Across two generations, we constructed a measure of SES by calculating the average of the standardized scores (z-scores) of education and income. This approach captures the independent contributions of each indicator to the overall construct of SES (Conger et al., 2021). When parents were around 44 years, we assessed SES by inquiring about their highest level of education and their household’s gross annual income (generation 1). When the children were around 27 years, we similarly assessed the second generation’s SES.

### 2.4. Home observations

At age 16/17, 45-min observations in the families’ homes were conducted to assess both material and immaterial investments. Two examiners visited the home and independently completed the Home Visitor Impressions Checklist (Oregon Social Learning Center, 1997; see also Dishion et al., 2004). The examiners’ ratings were averaged for each question, and these averages were used to compute the overall scores.

#### Material Investments.

Material investments were assessed using seven items scored on a 5-point scale (1 = not at all to 5 = very much so). These items included assessments of the neighborhood’s safety, signs of gang activity, the presence of a supportive community, the physical care of the children, cleanliness and tidiness of the living space, the presence of educational materials, and whether the living space was safe for children. The Cronbach’s Alpha for these items was .895 (Oregon Social Learning Center, 1997). The sum of these scores provided an overall measure of material investments, with higher scores indicating a greater presence of positive material investments in the home environment.

#### Immaterial Investments.

Family interactions were observed and coded to assess immaterial investments. This assessment included four facets: parental monitoring, positive relationship, rapport with family, and a deviancy score, all derived from the Coder Impressions Questionnaire (Dishion and Kavanagh, 2003). Each facet was measured on a 5-point scale (1 = not at all to 5 = very much so). Parental monitoring was assessed using six items (e.g., “Do parents set limits on child’s behavior). Positive relationship was based on ten items (e.g., Did the parent(s) praise or encourage the child?). Rapport with family was measured using twelve items (e.g., Were parents uncomfortable or threatened by questions?). The deviancy score was based on two items (e.g., Is the child affiliated with a deviant peer group?) and was reverse coded so that higher scores indicate lower deviancy. These four facets were combined to develop a latent construct for immaterial investments, with factor loadings ranging from .60 to .86.

#### Inhibitory control.

Inhibitory control was assessed using the inhibitory control subscale from the parent-rated Early Adolescent Temperament Questionnaire–Parent Report (EATQ) Revised (EATQ-R; Ellis and Rothbart, 2001). The inhibitory control subscale is an average score of eight items rated on a five-point Likert scale, with higher scores indicating greater inhibitory control. Each item has a 5-point Likert scale ranging from 1 (almost always untrue) to 5 (almost always true). Items include “My child can easily stop an activity when told to do so” and “My child has difficulty waiting in line”. We calculated a mean of the same construct measured at ages 22 and 23 to obtain a stable measure. Internal consistency for this subscale at each measurement was good (resp. α = 0.78 and α = 0.82).

### 2.5. Covariates

Children’s sex (47.2% females), age (at first measurement: Mean = 16.98, SD = .77), and race/ethnicity were included as covariates in the model. In total, 42.4% of respondents were European American, 29.2% were African American, and 28.4% were from various ethnic or racial groups, among which Latinx (6.8%), Asian American (5.2%), European-African American (3.5%), Native American (2.0%), Pacific Islander (.9%) and other (10%) (American Psychology Association, 2015). In the analyses, we dummy-coded race/ethnicity to represent European American (42.4%) and ethnically or racially minoritized individuals (57.6%). Data were part of a randomized controlled trial to test the effectiveness of the Family Check-Up. Therefore, we controlled for the intervention (i.e., who received the FCU [50.2%] versus control group [49.8%]).

### 2.6. Analyses

First, we computed descriptive statistics. Secondly, using Mplus version 7.4 (Muthen & Muthen, 1998–2017), we employed structural equation modeling to test the hypothesized path model. To determine model fit, we used the comparative fit index (CFI, Bentler, 1990), the Tucker Lewis Index (TLI, Kline, 1998), and the root mean squared estimate of approximation (RMSEA, Kline, 1998). Subsequently, using the Model Constraints methods, new latent variables were constructed to test whether SES of the first generation (i.e., family SES) would be indirectly linked to SES of the second generation via parental investments and inhibitory control (e.g., Muthén and Muthén, 2017; Otten et al., 2023).

### 2.7. Transparency and openness

The study’s design and its analysis were not pre-registered. Data supporting the findings of this study, along with the analysis code and other relevant materials can be found OSF Framework (Otten and Ha, 2024)

## 3. Results

### 3.1. Preliminary analyses

Table 1 shows the mean and standard deviation of key variables. Skewness (cutoff <2) and kurtosis (cutoff <7) of all variables fell within the acceptable range (see West et al., 1995). Regarding income, the median for the first generation fell between $30,000 and $39,999, while for the second generation, it ranged from $15,000 to $20,000. The observed differences in income between the first and second generations are likely attributable to differences in experience and career development. We conducted attrition analyses using all study variables to examine whether there was any systematic bias in the missing data. We found that none of these variables was related to missingness. Hence, maximum likelihood estimation under missing at random was used for the estimation of the model (Little and Rubin, 2002).

Table 2 presents the zero-order correlation coefficients between the main study variables. The results indicate that girls tended to have lower deviancy scores and reported higher levels of SES at age 27. Families with older children exhibited lower levels of material investments, monitoring, and rapport family scores, along with higher deviancy scores. Additionally, older children showed lower levels of second-generation SES. Children in the treatment group also had lower levels of second-generation SES. Being from an ethnically or racially minoritized group was associated with growing up in families with lower SES, fewer material investments, and lower levels of inhibitory control, as well as having lower SES themselves at age 27. All other correlations were consistent with expectations.

### 3.2. Findings of structural equation model

**Fig. 2.**
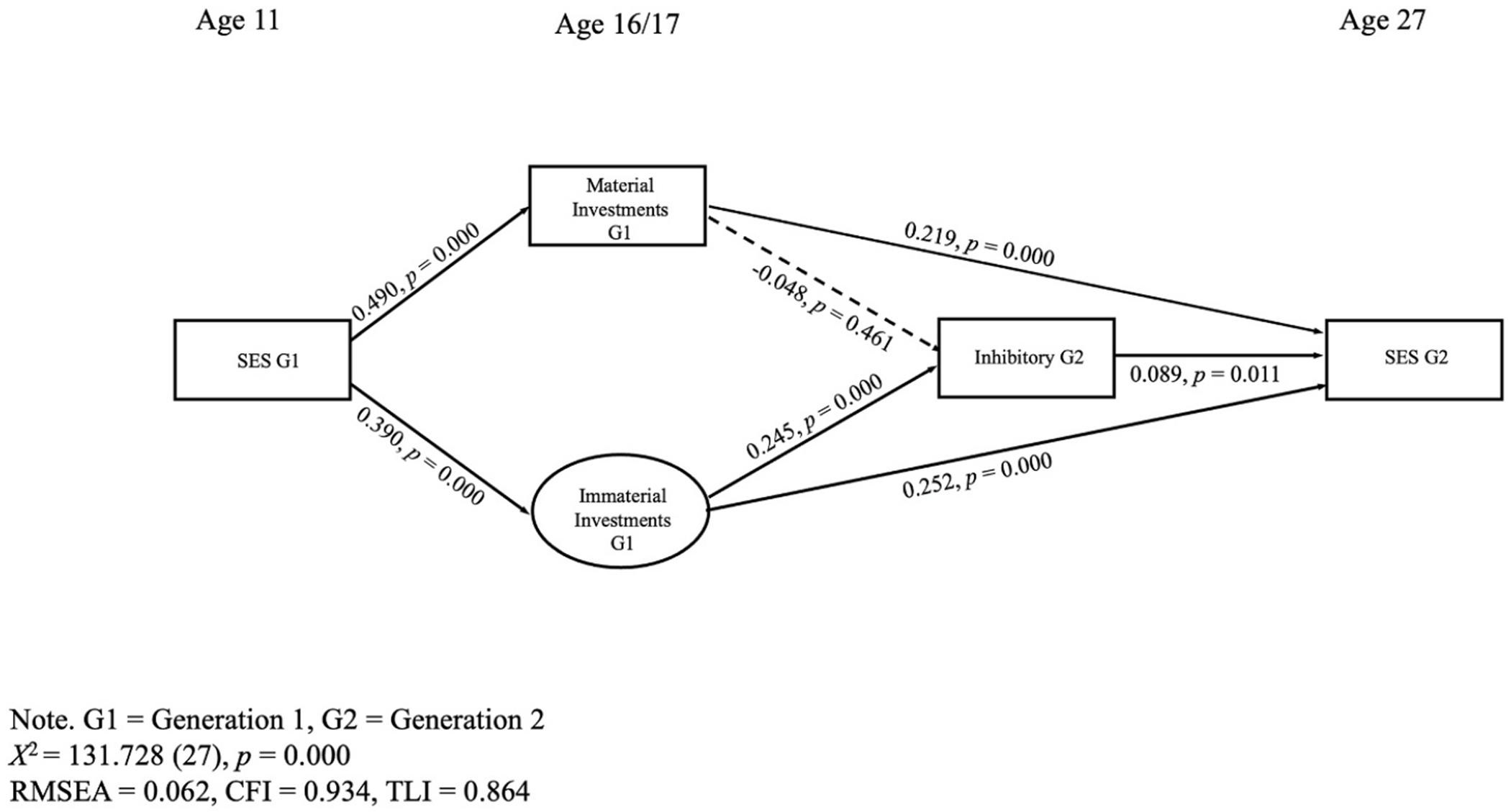
Path diagram illustrating the intergenerational transmission of socioeconomic status (SES). The model delineates the relationships between SES in the first generation (G1) at age 16/17 and the SES in the second generation (G2) at age 27, partially mediated by material and immaterial investments at G1 and inhibitory control at G2. Solid lines represent significant paths with standardised coefficients and p-values, while the dashed line indicates a non-significant path. Factor loadings for the measurement of the latent constructs are provided in the text. Model fit indices are included in the note below the diagram.

Fig. 2 presents the findings of the structural equation model. Model fit indices suggested a sufficient fit to the data, *χ*^2^ (27) = 131.728, *p* = 0.000; CFI = .934; TLI = .864; RMSEA = .062. Consistent with the Interactionist Model, Family SES was positively associated with higher levels of Parental Immaterial and Material Investments (resp. *β* = .390, *p* < 0.000 and *β* = .490, *p* < 0.000). Immaterial Investments were positively related to inhibitory control (*β* = .245, *p* < 0.000), but Material Investments were not (*β* = −.048, *p* = 0.461). Immaterial and Material investments were positively associated with SES in the next generation (resp. *β* = .252, *p* = 0.000 and *β* = .219, *p* = 0.000). Finally, inhibitory control was associated with higher levels of SES in the second generation (*β* = .089, *p* = 0.011).

Note that covariates were included in the analysis but not in the figure. Older participants (*r* = −.138, *p* = 0.001) and ethnically or racially minoritized participants (*r* = .394, *p* = 0.000) were more likely from families with lower SES. Older children reported less material and immaterial investments (resp. *β* = −.080, *p* = 0.035 and *β* = −.144, *p* = 0.001). Moreover, ethnically or racially minoritized children reported more parental immaterial investments (*β* = .111, *p* = 0.011), but less inhibitory control (*β* = .100, *p* = 0.004). Female participants (*β* = .138, *p* = 0.000), white participants (*β* = −.141, *p* = 0.000), and adolescents in the control condition (*β* = −.065, *p* = 0.046) were likely to report higher levels of SES (second generation). SES of the second generation has an explained variance of .293, meaning that 29.3% of its variance is explained by the model.

### 3.3. Indirect effects

There were significant indirect effects from family SES on SES in the next generation via material investments (*β* = .108; 95% *CI* = .040, .172, *p* = 0.001); via immaterial investments (*β* = .099; 95% *CI* = .039, .160, *p* = 0.001); and via immaterial investments and inhibitory control (*β* = .009; 95% *CI* = .001, .017, *p* = 0.035). Please note that the direct link between family SES and SES of the second generation was not included in the conceptual model we tested. If this direct link had been incorporated, that link was significant (i.e., *β* = .312, *p* = 0.000), however the direct link between material investments and SES of the next generation, as well as the indirect effect of family SES on the SES of the second generation through material investments would have disappeared. All other effects remained unchanged. Additionally, the (insignificant) link between family SES and inhibitory control was excluded to maintain the model’s parsimony.

## 4. Discussion

Responsive to the national call to combat SES inequities, we studied a modifyable underlying psychological mechanisms of the intergenerational transmission of SES as potential key targets for prevention and intervention. Specifically, we investigated parental material and immaterial investments and adolescents’ inhibitory control as underlying psychological mechanism. As hypothesized, we found intergenerational transmission of SES. Adolescents who grew up in low SES households were more likely to experience low SES as adults. Importantly, we found evidence for parental immaterial and material investments as underlying mechanisms. Lower SES was associated with less parental immaterial and material investments, which associated with lower SES in the next generation. Another critical mechanism was through adolescents’ inhibitory control. Specifically, suggesting partial mediation, SES showed an indirect association with the SES of the next generation via immaterial parental investments and adolescents’ inhibitory control. In other words, lower levels of parental SES were associated with strained parent-child relationships, which in turn related to adolescents’ inhibitory control, which ultimately associated with lower SES in the next generation. It is important to note that no association was found between material investments and adolescents’ inhibitory control.

Parental immaterial investments playing a more critical role in the development of inhibitory control, - which is one aspect of self-regulation-, than material investments (Muller et al., 2013) is in line with research showing that supportive parental interactions, emotional support, and self-regulation strategies cultivate inhibitory control (Mun et al., 2019). It underscores that inhibitory control is a psychological construct closely related to neural development and cognitive processes that vary by levels of positive parenting and is not directly influenced by material resources (e.g., Constantinidis and Luna, 2019). The lack of association between material investments and inhibitory control could also stem from how the different constructs were measured in our study. For instance, inhibitory control was assessed through a questionnaire, which may not fully capture the nuances of how material resources impact inhibitory control (Petersen et al., 2016).

The inclusion of a direct effect of family SES on SES of the next generation makes the effect of material investments nonsignificant, but the indirect effects of immaterial investments and inhibitory control remain significant. This suggests that the hypothesis—that family SES relates to SES of the next generation through underlying parental and psychological processes—still holds. While we have focused on the psychological and parental investment pathways in our preferred model, we acknowledge that family SES and SES of the next generation may also be linked by additional factors not captured in our study. These factors could include broader socio-cultural dynamics, access to social capital, or community resources. Such broader perspective aims to provide a more comprehensive understanding of the complexities involved in the transmission of SES across generations.

Concerning the covariates included in the model, some of the correlations that were significant bivariately were no longer significant in the overall model, or became significant in the unexpected direction. Specfically, we found lower material investments in ethnically or racially minoritized individuals when looking at bivariate correlations. However, in the SEM-model – when controlling for family SES - ethnically or racially minoritized families showed *more* immaterial investments. There are several possible explanations. The strong emphasis on family and community ties may be one explanation. Familism, for example, is a key cultural value for Hispanic/Latino families and emphasizes the importance of close-knit family relationships and loyalty, honor, and obligation to the family, promoting positive parent child relationships (Cahill et al., 2021). This emphasis on strong family bonds can lead to greater immaterial investments in children as a way of fostering resilience and ensuring the well-being of the next generation (Hughes et al., 2006). Moreover, some research suggests that parents in racially minoritized groups may engage in compensatory behaviors to buffer their children from the effects of systemic inequalities and discrimination. In this context, parents might increase their immaterial investments, such as monitoring and positive parenting practices, as a protective mechanism to help their children navigate challenging social environments (Pittman & Chase-Lansdale, 2001). In addition, racially minoritized groups often rely on strong social networks as a form of social capital. These networks can provide emotional, informational, and instrumental support that reinforces immaterial investments in children. Parents in these communities may place a higher value on building rapport and maintaining positive relationships within the family as a means of leveraging this social capital to support their children’s development (McLoyd, 1990). Future research should focus on this finding as it may reflect a specific cultural phenomenom that we were not able to capture in this study. However, there may also be an methodological explanation. For instance, a structural equation model can show significant pathways or correlations that are not supported by bivariate correlations. This can occur due to the nature of SEM and the multivariate relationships it examines, which can reveal relationships that are not evident in bivariate correlations (Hair et al., 2021). Moreover, control variables can alter the relationships between variables and lead to different results than simple bivariate correlations.

The present study has several notable limitations, particularly in its methodology. Firstly, in this study, we conceptualized SES as a formative construct, with education and income as independent contributors to the construct. This approach differs from treating SES as a reflective construct, where SES is seen as an underlying latent variable that influences observable indicators like education and income. Using SES in a formative manner allows each indicator to contribute uniquely, but it also means we might not fully capture the broader socio-economic context that SES encompasses. SES is more than just income and education; it includes aspects such as wealth, occupational status, neighborhood quality, and access to social and cultural capital. By focusing only on these two indicators, we may overlook other significant dimensions of SES that could influence our outcomes. Future research could benefit from considering both formative and reflective models of SES to understand how these various elements interact and collectively impact socio-economic status. This approach would provide a more nuanced understanding of SES and its complex role in shaping life outcomes across generations (Antonoplis, 2023). Secondly, although the EATQ-R is commonly used from late childhood through late adolescence (18–24 years of age) (Snyder et al., 2015), the utilization of this instrument, originally designed for early to mid-adolescents, to assess inhibitory control of 22-year-old participants may be limitation, given potential developmental and contextual mismatches. This discrepancy may impact the validity of its findings for this age group, suggesting the need for measures tailored to late adolescence or early adulthood in future research. Thirdly, the operationalization of material investments may not reflect the changes in the importance of varying material investments over time. Fourthly, in this study we concentrated on inhibitory control. We did not examine any other psychological processes that may function as potential mediators of the association between material/immaterial investments and SES. This makes it also difficult to evaluate whether the effect that we found is specific to inhibitory control versus other related temperament or executive function processes. Fifthly, given the limited sample size we strived to test a model that included only the most important theoretical parameters following the Interactionist Model. However, ideally, multigenerational research would start at an early age and include more than two generations. Finally, the current study did not test differences between ethnically and racially minoritized and White participants but instead included it as a covariate in the analyses. Furthermore, we combined all ethnic/racial minority groups. Economic inequality is interwoven with the intersectionality of race, class, and sex or gender (Cole, 2009; Syed and Ajayi, 2018), but we could not investigate the current intergenerational SES model through an intersectionality lens. We acknowledge that differences exist between various ethnic/racial minority groups. African Americans, in particular, are behind in their intergenerational income mobility due to historical experiences with slavery and historical and current segregation and discrimination (Hardaway and McLoyd, 2009). Future research with larger sample sizes for ethnic/racial minority groups would provide opportunities to investigate the unique effects for African Americans, Latinx/Hispanic, Native American, and Asian Americans.

### 4.1. Implications

The findings of our study provide important input to the current discussion on equity and equality issues. Understanding the psychological mechanisms in transgenerational SES can help identify potential interventions and policies to break this cycle and promote upward mobility. In this study, we emphasized the psychological pathway in which first-generation SES influences immaterial investments, affecting inhibitory control and, ultimately, second-generation SES. However, our findings indicate that the strongest link between first- and second-generation SES is through material investments. Material resources, such as quality education, safe housing, and economic support, directly shape a child’s opportunities and life outcomes, contributing significantly to upward social mobility (Conger et al., 2010; Gershoff et al., 2007; Kaushal et al., 2011; Kalil and Ryan, 2020). While immaterial investments like parental support are crucial for psychological development, material investments provide the foundational resources necessary for success (e.g., Conger et al., 2012). These findings highlight the need to consider both material and immaterial investments in understanding intergenerational SES transmission.

By enhancing access to material resources, such as quality education, stable housing, and supportive community environments, foundation is created that allows for healthier psychological development and more effective parental investments. These improvements can, in turn, make it easier to address the psychological aspects through targeted interventions, such as parent education programs, that build on a more secure material and immaterial base. The knowledge of the different processes involved in transgenerational SES can inform the development of effective public policies. Policies can be designed to address the difference in material and immaterial investments in low SES families or the specific psychological barriers that hinder individuals from low SES backgrounds, such as lack of self-efficacy, limited aspirations, or negative stereotypes. This can lead to targeted community interventions that support educational and economic opportunities for disadvantaged individuals. Understanding how psychological factors influence the transmission of SES across generations can guide the development of educational and parenting interventions. This research can inform the design of early intervention programs that focus on improving children’s cognitive and emotional skills from an early age. Such programs can help level the playing field and provide all children with a stronger foundation for success. Knowledge of the psychological mechanisms involved in transgenerational SES can highlight factors contributing to resilience among disadvantaged individuals.

## 5. Conclusion

While the fraction of children who earn more than their parents has declined over time (Chetty et al., 2017), there is still a strong link between SES across generations. Being born into families that are more privileged in terms of wealth, education, and social standing provides advantages in life domains such as health, cognitive abilities, and socio-emotional well-being (e.g., Bradley and Corwyn, 2002; Evans and Kim, 2013). This contributes to the intergenerational stability of SES, in which children who are raised in more educated and privileged families are more likely to achieve similar advantages themselves. Our study shows that inhibitory control is an important construct in the processes of intergenerational transmission of SES. However, inhibitory control is just one potential mechanism. Gaining more knowledge about the psychological processes underlying transgenerational levels of SES is essential for addressing social inequalities, promoting social mobility, and creating a more equitable society. It provides the foundation for evidence-based policies and interventions that empower individuals and families to overcome barriers and achieve their full potential.

However, in order to address the material and immaterial inequities and psychological mechanisms, larger structural changes are needed. Specifically, enhancing access to quality education, providing supportive school environments, and implementing policies that ensure equitable resource distribution can have a significant impact. Additionally, political advocacy aimed at creating policies that support low-SES families, such as increasing minimum wage, improving housing conditions, and ensuring access to healthcare, is essential for creating a more equitable society. Hence, we believe that while intervention programs are valuable, they must be part of a broader strategy that includes systemic change to effectively reduce inequities.

## Figures and Tables

**Table 1:** Descriptive statistics.

Categorical Variables		Percentages

1. Sex	Male (0)	52.8%
	Female (1)	47.2%
2. Ethnicity	European American (0)	42.4%
	Other (1)	57.6%
3. Treatment	Treatment condition (0)	50.2%
	Control condition (1)	49.8%
4. Education G1	Anything up to High School	11.1%
	High School	22.7%
	Some College/Associate Degree	40.6%
	College	11.4%
	Graduate School	14.2%
5. Education G2	Anything up to High School	8.7%
	High School	20.7%
	Some College/Associate Degree	43.9%
	College	21.2%
	Graduate School	5.4%
		
Continuous Variables		Means (SD)
	
6. Age (in months)		203.82 (9.188)
7. Material Investments		30.674 (5.290)
8. Parental Monitoring		21.546 (3.569)
9. Positive Relationship		43.397 (4.938)
10. Rapport with Family Score		50.566 (5.374)
11. Deviant Talk Parents		8.174 (1.741)
12. Inhibitory Control Age 22/23		3.969 (.520)

**Table 2:** Bivariate correlations.

	1.	2.	3.	4.	5.	6.	7.	8.	9.	10.	11.
1. Sex	–										
2. Age	−.034	–									
3. Ethnicity	.016	.097**	–								
4. Treatment	.017	.033	.008	–							
5. SES Generation 1	.006	−.148**	−.385**	−.071	–						
6. Material Investments	.015	−.156**	−.218**	−.023	.496**	–					
7. Parental Monitoring	−.011	−.222**	−.067	.000	.311**	.545**	–				
8. Positive Relationship	.023	−.058	−.009	−.002	.160**	.320**	.469**	–			
9. Rapport with Family Score	.039	−.096*	−.057	−.015	.244**	.459**	.516**	.731**	–		
10. Deviancy Score	−.151**	.073*	.005	.025	−.160**	−.397**	−.477**	−.464**	−.459**	–	
11. Inhibitory Control Age 22/23	.023	−.053	−.104*	.000	.087*	.138*	.139**	.159**	.138**	.179**	–
12. SES Generation 2	.166**	−.159**	−.213**	−.074*	.465**	.414**	.319**	.209**	.250**	.341**	.194**

Note. p < 0.05, p < 0.01. Dummy-coded variables: Sex (0 = male, 1 = female); Ethnicity (0 = European Americans, 1 = ethnically/racially minoritized individuals);

Treatment (0 = control group, 1 = intervention group).

*Note*.

* = *p* < 0.05

** = *p* < 0.001.
